# (*E*)-3-(2-Hydr­oxy-4-methoxy­benzyl­idene­amino)benzonitrile

**DOI:** 10.1107/S1600536809023174

**Published:** 2009-06-20

**Authors:** Jian-Cheng Zhou, Zheng-Yun Zhang, Chuan-Ming Zhang, Nai-Xu Li

**Affiliations:** a College of Chemistry and Chemical Engineering, Southeast University, Nanjing 211189, People’s Republic of China

## Abstract

In the mol­ecule of the title compound, C_15_H_12_N_2_O_2_, the aromatic rings are oriented at a dihedral angle of 28.11 (3)°. Intra­molecular O—H⋯N hydrogen bonding results in the formation of a planar six-membered ring, which is nearly coplanar with the adjacent ring at a dihedral angle of 1.53 (3)°. In the crystal structure, π–π contacts between the benzene rings [centroid–centroid distance = 3.841 (1) Å] may stabilize the structure.

## Related literature

For general background, see: Chen *et al.* (2008 ▶); May *et al.* (2004 ▶); Weber *et al.* (2007 ▶). For bond-length data, see: Allen *et al.* (1987 ▶).
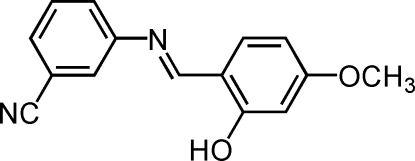


## Experimental

### 

#### Crystal data

C_15_H_12_N_2_O_2_
*M* *_r_* = 252.27
Monoclinic, 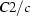

*a* = 14.484 (3) Å
*b* = 6.6587 (13) Å
*c* = 26.461 (5) Å
β = 102.14 (3)°
*V* = 2494.9 (9) Å^3^
*Z* = 8
Mo *K*α radiation
μ = 0.09 mm^−1^
*T* = 294 K
0.2 × 0.2 × 0.2 mm

#### Data collection

Rigaku SCXmini diffractometer
Absorption correction: multi-scan (*CrystalClear*; Rigaku, 2005 ▶) *T*_min_ = 0.982, *T*_max_ = 0.982
12044 measured reflections
2861 independent reflections
1608 reflections with *I* > 2σ(*I*)
*R*_int_ = 0.062

#### Refinement

*R*[*F* ^2^ > 2σ(*F* ^2^)] = 0.059
*wR*(*F* ^2^) = 0.142
*S* = 1.01
2861 reflections
176 parameters
H atoms treated by a mixture of independent and constrained refinement
Δρ_max_ = 0.14 e Å^−3^
Δρ_min_ = −0.18 e Å^−3^

### 

Data collection: *CrystalClear* (Rigaku, 2005 ▶); cell refinement: *CrystalClear*; data reduction: *CrystalClear*; program(s) used to solve structure: *SHELXS97* (Sheldrick, 2008 ▶); program(s) used to refine structure: *SHELXL97* (Sheldrick, 2008 ▶); molecular graphics: *ORTEP-3 for Windows* (Farrugia, 1997 ▶) and *PLATON* (Spek, 2009 ▶); software used to prepare material for publication: *SHELXL97* and *PLATON*.

## Supplementary Material

Crystal structure: contains datablocks I, global. DOI: 10.1107/S1600536809023174/hk2714sup1.cif

Structure factors: contains datablocks I. DOI: 10.1107/S1600536809023174/hk2714Isup2.hkl

Additional supplementary materials:  crystallographic information; 3D view; checkCIF report

## Figures and Tables

**Table 1:** Hydrogen-bond geometry (Å, °)

*D*—H⋯*A*	*D*—H	H⋯*A*	*D*⋯*A*	*D*—H⋯*A*
O1—H1*B*⋯N1	0.92 (3)	1.76 (3)	2.592 (3)	149 (3)

**Fig. 1.**
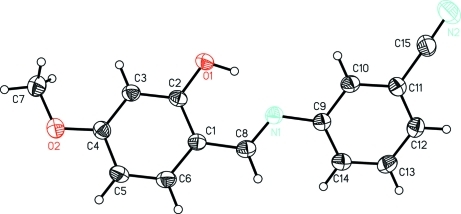
The molecular structure of the title molecule, with the atom-numbering scheme. Hydrogen bond is shown as a dashed line.

## supplementary crystallographic information

### Comment

Schiff base compounds have received considerable attention for many years, primarily due to their importance in the development of coordination chemistry related to magnetism (Weber *et al.*, 2007), catalysis (Chen *et al.*, 2008) and biological process (May *et al.*, 2004). Our group is interested in the syntheses and preparation of Schiff bases. We report herein the synthesis and crystal structure of the title compound.

In the molecule of the title compound (Fig 1), the bond lengths (Allen *et al.*, 1987) and angles are within normal ranges. Rings A (C1-C6) and B (C9-C14) are, of course, planar and they are oriented at a dihedral angle of 28.11 (3)°. Atoms O1, O2, N1, C7 and C8 are 0.005 (3), 0.014 (3), 0.067 (3), 0.100 (3) and 0.054 (3) Å away from the plane of ring A, while atoms N1, N2 and C15 are 0.053 (3), 0.002 (3) and 0.002 (3) Å away from the plane of ring B, respectively. Intramolecular O-H···N hydrogen bond (Table 1) results in the formation of a planar six-membered ring C (O1/N1/C1/C2/C8/H1B), which is oriented with respect to rings A and B at dihedral angles of A/C = 1.53 (3) and B/C = 27.66 (3) °. So, rings A and C are nearly coplanar.

In the crystal structure, the π–π contact between the benzene rings, Cg1—Cg2^i^, [symmetry code: (i) -x, y, 1/2 - z, where Cg1 and Cg2 are centroids of the rings A (C1-C6) and B (C9-C14), respectively] may stabilize the structure, with centroid-centroid distance of 3.841 (1) Å.

### Experimental

For the preparation of the title compound, 3-aminobenzonitrile (472 mg, 4 mmol) and 2-hydroxy-4-methoxybenzaldehyde (608 mg, 4 mmol) were dissolved in ethanol (20 ml). The mixture was heated to reflux for 5 h, and then cooled to room temperature. The solution was filtered and after two weeks, yellow crystals suitable for X-ray analysis were obtained (yield; 85%).

### Refinement

H atom (for OH) was located in difference Fourier map and refined isotropically. The remaining H atoms were positioned geometrically, with C-H = 0.93 and 0.96 Å for aromatic and methyl H, respectively, and constrained to ride on their parent atoms, with U_iso_(H) = xU_eq_(C), where x = 1.5 for methyl H and x = 1.2 for aromatic H atoms.

### Crystal data

**Table:**

C_15_H_12_N_2_O_2_	*F*(000) = 1056
*M**_r_* = 252.27	*D*_x_ = 1.343 Mg m^−^^3^
Monoclinic, *C*2/*c*	Mo *K*α radiation, λ = 0.71073 Å
Hall symbol: -C 2yc	Cell parameters from 9417 reflections
*a* = 14.484 (3) Å	θ = 3.1–27.7°
*b* = 6.6587 (13) Å	µ = 0.09 mm^−^^1^
*c* = 26.461 (5) Å	*T* = 294 K
β = 102.14 (3)°	Prism, yellow
*V* = 2494.9 (9) Å^3^	0.2 × 0.2 × 0.2 mm
*Z* = 8	

### Data collection

**Table:**

Rigaku SCXmini diffractometer	2861 independent reflections
Radiation source: fine-focus sealed tube	1608 reflections with *I* > 2σ(*I*)
graphite	*R*_int_ = 0.062
Detector resolution: 13.6612 pixels mm^-1^	θ_max_ = 27.5°, θ_min_ = 3.2°
ω scans	*h* = −18→18
Absorption correction: multi-scan (*CrystalClear*; Rigaku, 2005)	*k* = −8→8
*T*_min_ = 0.982, *T*_max_ = 0.982	*l* = −34→34
12044 measured reflections	

### Refinement

**Table:**

Refinement on *F*^2^	Primary atom site location: structure-invariant direct methods
Least-squares matrix: full	Secondary atom site location: difference Fourier map
*R*[*F*^2^ > 2σ(*F*^2^)] = 0.059	Hydrogen site location: inferred from neighbouring sites
*wR*(*F*^2^) = 0.142	H atoms treated by a mixture of independent and constrained refinement
*S* = 1.01	*w* = 1/[σ^2^(*F*_o_^2^) + (0.0618*P*)^2^ + 0.0432*P*] where *P* = (*F*_o_^2^ + 2*F*_c_^2^)/3
2861 reflections	(Δ/σ)_max_ < 0.001
176 parameters	Δρ_max_ = 0.14 e Å^−^^3^
0 restraints	Δρ_min_ = −0.18 e Å^−^^3^

### Special details

**Table:**

Geometry. All e.s.d.'s (except the e.s.d. in the dihedral angle between two l.s. planes) are estimated using the full covariance matrix. The cell e.s.d.'s are taken into account individually in the estimation of e.s.d.'s in distances, angles and torsion angles; correlations between e.s.d.'s in cell parameters are only used when they are defined by crystal symmetry. An approximate (isotropic) treatment of cell e.s.d.'s is used for estimating e.s.d.'s involving l.s. planes.
Refinement. Refinement of *F*^2^ against ALL reflections. The weighted *R*-factor *wR* and goodness of fit *S* are based on *F*^2^, conventional *R*-factors *R* are based on *F*, with *F* set to zero for negative *F*^2^. The threshold expression of *F*^2^ > σ(*F*^2^) is used only for calculating *R*-factors(gt) *etc*. and is not relevant to the choice of reflections for refinement. *R*-factors based on *F*^2^ are statistically about twice as large as those based on *F*, and *R*- factors based on ALL data will be even larger.

### Fractional atomic coordinates and isotropic or equivalent isotropic displacement parameters (Å^2^)

**Table:**

	*x*	*y*	*z*	*U*_iso_*/*U*_eq_	
O1	0.60788 (11)	−0.3053 (2)	0.21473 (6)	0.0585 (4)	
H1B	0.602 (2)	−0.228 (4)	0.2426 (12)	0.116 (11)*	
O2	0.70583 (11)	−0.2132 (2)	0.05513 (6)	0.0605 (4)	
N1	0.61594 (10)	0.0051 (2)	0.27458 (6)	0.0452 (4)	
N2	0.56309 (17)	−0.2127 (4)	0.47862 (8)	0.0890 (8)	
C1	0.66562 (12)	0.0183 (3)	0.19441 (7)	0.0415 (5)	
C2	0.64330 (13)	−0.1851 (3)	0.18223 (7)	0.0426 (5)	
C3	0.65629 (13)	−0.2662 (3)	0.13598 (7)	0.0458 (5)	
H3A	0.6414	−0.4001	0.1282	0.055*	
C4	0.69136 (13)	−0.1477 (3)	0.10142 (7)	0.0456 (5)	
C5	0.71476 (14)	0.0530 (3)	0.11294 (8)	0.0537 (6)	
H5A	0.7387	0.1320	0.0897	0.064*	
C6	0.70216 (14)	0.1324 (3)	0.15864 (8)	0.0506 (5)	
H6A	0.7183	0.2658	0.1662	0.061*	
C7	0.67817 (16)	−0.4139 (4)	0.03934 (8)	0.0657 (7)	
H7A	0.6926	−0.4398	0.0062	0.099*	
H7B	0.6115	−0.4292	0.0371	0.099*	
H7C	0.7118	−0.5072	0.0642	0.099*	
C8	0.64973 (12)	0.1077 (3)	0.24112 (7)	0.0459 (5)	
H8A	0.6641	0.2427	0.2475	0.055*	
C9	0.59329 (12)	0.0935 (3)	0.31897 (7)	0.0425 (5)	
C10	0.59023 (13)	−0.0336 (3)	0.35993 (7)	0.0465 (5)	
H10A	0.6050	−0.1687	0.3575	0.056*	
C11	0.56549 (13)	0.0373 (3)	0.40448 (7)	0.0465 (5)	
C12	0.54203 (14)	0.2386 (3)	0.40845 (8)	0.0530 (6)	
H12A	0.5251	0.2869	0.4382	0.064*	
C13	0.54431 (14)	0.3653 (3)	0.36748 (8)	0.0553 (6)	
H13A	0.5288	0.5001	0.3697	0.066*	
C14	0.56938 (14)	0.2945 (3)	0.32322 (8)	0.0504 (5)	
H14A	0.5703	0.3820	0.2959	0.060*	
C15	0.56360 (16)	−0.1001 (4)	0.44628 (9)	0.0607 (6)	

### Atomic displacement parameters (Å^2^)

**Table:**

	*U*^11^	*U*^22^	*U*^33^	*U*^12^	*U*^13^	*U*^23^
O1	0.0842 (11)	0.0452 (9)	0.0495 (9)	−0.0166 (8)	0.0219 (8)	0.0008 (8)
O2	0.0731 (10)	0.0597 (10)	0.0543 (9)	−0.0061 (8)	0.0262 (8)	−0.0050 (8)
N1	0.0460 (10)	0.0446 (10)	0.0429 (10)	−0.0022 (8)	0.0045 (8)	−0.0012 (8)
N2	0.1156 (19)	0.0890 (17)	0.0664 (14)	0.0167 (14)	0.0283 (13)	0.0222 (13)
C1	0.0390 (10)	0.0397 (11)	0.0441 (11)	−0.0004 (9)	0.0052 (8)	0.0032 (9)
C2	0.0418 (11)	0.0420 (12)	0.0421 (11)	−0.0008 (9)	0.0043 (9)	0.0072 (10)
C3	0.0477 (12)	0.0408 (12)	0.0488 (12)	−0.0056 (9)	0.0102 (9)	−0.0002 (10)
C4	0.0418 (12)	0.0491 (13)	0.0467 (12)	0.0012 (9)	0.0110 (9)	0.0026 (10)
C5	0.0596 (13)	0.0461 (13)	0.0598 (14)	−0.0060 (10)	0.0229 (11)	0.0076 (11)
C6	0.0508 (13)	0.0407 (12)	0.0614 (14)	−0.0057 (9)	0.0139 (10)	0.0008 (11)
C7	0.0796 (17)	0.0624 (16)	0.0582 (15)	−0.0047 (13)	0.0213 (12)	−0.0105 (12)
C8	0.0399 (11)	0.0435 (12)	0.0511 (12)	−0.0048 (9)	0.0027 (9)	−0.0005 (10)
C9	0.0373 (11)	0.0436 (12)	0.0441 (11)	−0.0021 (9)	0.0026 (8)	−0.0008 (10)
C10	0.0465 (12)	0.0403 (11)	0.0510 (12)	0.0030 (9)	0.0068 (9)	0.0018 (10)
C11	0.0416 (11)	0.0531 (13)	0.0439 (12)	−0.0005 (9)	0.0069 (9)	0.0020 (10)
C12	0.0518 (13)	0.0569 (14)	0.0517 (13)	−0.0007 (11)	0.0144 (10)	−0.0087 (11)
C13	0.0558 (14)	0.0440 (13)	0.0675 (15)	0.0019 (10)	0.0161 (11)	−0.0056 (12)
C14	0.0500 (13)	0.0450 (12)	0.0542 (13)	−0.0012 (10)	0.0069 (10)	0.0048 (11)
C15	0.0658 (15)	0.0659 (16)	0.0515 (14)	0.0083 (12)	0.0147 (11)	0.0030 (13)

### Geometric parameters (Å, °)

**Table:**

O1—C2	1.352 (2)	C7—H7B	0.9600
O1—H1B	0.92 (3)	C7—H7C	0.9600
O2—C4	1.357 (2)	C8—C1	1.434 (3)
O2—C7	1.432 (3)	C8—H8A	0.9300
N1—C8	1.293 (2)	C9—C10	1.383 (3)
N1—C9	1.413 (2)	C9—C14	1.393 (3)
C1—C6	1.402 (2)	C10—C11	1.385 (3)
C2—C1	1.414 (3)	C10—H10A	0.9300
C3—C2	1.386 (3)	C11—C12	1.392 (3)
C3—C4	1.383 (3)	C11—C15	1.440 (3)
C3—H3A	0.9300	C12—C13	1.380 (3)
C4—C5	1.397 (3)	C12—H12A	0.9300
C5—H5A	0.9300	C13—H13A	0.9300
C6—C5	1.367 (3)	C14—C13	1.380 (3)
C6—H6A	0.9300	C14—H14A	0.9300
C7—H7A	0.9600	C15—N2	1.139 (3)
			
C2—O1—H1B	107.1 (18)	H7A—C7—H7C	109.5
C4—O2—C7	118.43 (16)	H7B—C7—H7C	109.5
C8—N1—C9	122.42 (18)	N1—C8—C1	121.54 (19)
C2—C1—C8	121.61 (17)	N1—C8—H8A	119.2
C6—C1—C2	117.77 (17)	C1—C8—H8A	119.2
C6—C1—C8	120.60 (19)	C10—C9—N1	116.60 (18)
O1—C2—C1	121.40 (17)	C10—C9—C14	118.36 (18)
O1—C2—C3	118.10 (18)	C14—C9—N1	124.96 (18)
C3—C2—C1	120.50 (17)	C9—C10—H10A	119.5
C2—C3—H3A	120.0	C11—C10—C9	121.01 (19)
C4—C3—C2	119.91 (19)	C11—C10—H10A	119.5
C4—C3—H3A	120.0	C10—C11—C12	120.22 (19)
O2—C4—C3	124.17 (19)	C10—C11—C15	119.15 (19)
O2—C4—C5	115.32 (18)	C12—C11—C15	120.63 (19)
C3—C4—C5	120.51 (19)	C11—C12—H12A	120.6
C4—C5—H5A	120.3	C13—C12—C11	118.87 (19)
C6—C5—C4	119.45 (19)	C13—C12—H12A	120.6
C6—C5—H5A	120.3	C12—C13—C14	120.8 (2)
C1—C6—H6A	119.1	C12—C13—H13A	119.6
C5—C6—C1	121.8 (2)	C14—C13—H13A	119.6
C5—C6—H6A	119.1	C9—C14—H14A	119.6
O2—C7—H7A	109.5	C13—C14—C9	120.7 (2)
O2—C7—H7B	109.5	C13—C14—H14A	119.6
O2—C7—H7C	109.5	N2—C15—C11	178.2 (3)
H7A—C7—H7B	109.5		

### Hydrogen-bond geometry (Å, °)

**Table:**

*D*—H···*A*	*D*—H	H···*A*	*D*···*A*	*D*—H···*A*
O1—H1B···N1	0.92 (3)	1.76 (3)	2.592 (3)	149 (3)
